# Structure and Microhardness of Cu-Ta Joints Produced by Explosive Welding

**DOI:** 10.1155/2013/256758

**Published:** 2013-12-23

**Authors:** Iu. N. Maliutina, V. I. Mali, I. A. Bataev, A. A. Bataev, M. A. Esikov, A. I. Smirnov, K. A. Skorokhod

**Affiliations:** ^1^Novosibirsk State Technical University, Karl Marx Prospect 20, 630073 Novosibirsk, Russia; ^2^Lavrentyev Institute of Hydrodynamics, Siberian Branch, Russian Academy of Sciences, Akademika Lavrentyev Prospect 15, 630090 Novosibirsk, Russia

## Abstract

The structure and microhardness of Cu-Ta joints produced by explosive welding were studied. It was found that, during explosive welding, an intermediate layer 20⋯40 **μ**m thick with a finely dispersed heterophase structure, formed between the welded copper and tantalum plates. The structure of the layer was studied by scanning and transmission electron microscopy. Microvolumes with tantalum particles distributed in a copper matrix and microvolumes of copper particles in a tantalum matrix were detected. The tantalum particles in copper have a size of 5⋯500 nm, with a predominance of 5⋯50 nm particles. A mechanism for the formation of the finely dispersed heterophase structure in explosive welding is proposed. The microhardness of interlayers with the heterophase structure reaches 280 HV, which far exceeds the microhardness of copper (~130 HV) and tantalum (~160 HV). Many twins of deformation origin were found in the structure of the copper plate. The effect of heating temperature in the range from 100 to 900°C on the microhardness of copper, tantalum, and the Cu-Ta welded joint was studied. Upon heating to 900°C, the microhardness of the intermediate layer decreases from 280 to 150 HV. The reduction in the strength properties of the weld material is mainly due to structural transformations in copper.

## 1. Introduction

One of the most essential problems of modern materials science is the development of reliable joints of dissimilar metals with significantly different physical and mechanical properties [1–3]. There are a number of efficient methods for producing composites of this type based on fusion welding. Welding of reactive metals often involves the formation of brittle phases, for example, intermetallic compounds, in the fused joint. This problem is encountered in joining of nickel and aluminum, aluminum and titanium, titanium and iron, and many others materials [4, 5]. One of the most effective methods for preventing the formation of brittle chemical compounds is the application of diffusion barriers based on refractory metals, such as Ta, Nb, V, and W. Due to the large difference in physicochemical and mechanical properties between the welded dissimilar metals and refractory barriers, in some cases it is reasonable to produce intermediate barriers in the form of inserts by joining refractory metals with copper [6–8].

Below we consider in detail a Cu-Ta system characterized by nearly zero mutual solubility of the components in the solid state [9, 10] and high structural stability and mechanical strength of the joint at elevated temperature. Wang et al. [11] note the absence of structural transformations and changes in mechanical properties of a sputter-deposited Cu-Ta composite during heating at 900°C for 100 h. Greenberg et al. [12, 13] have shown that explosive welding of materials with no mutual solubility results in the formation of a heterophase mixture in the joint region with particle sizes of the dispersed phase similar to those of colloids.

This paper deals with the structure and microhardness of welds produced by explosive welding of copper and tantalum plates followed by heat treatment.

## 2. Materials and Methods

For explosive welding we used plates of technically pure copper (99.98% Cu) and tantalum (99.95% Ta). The materials used are significantly different in structure, density, melting point, and thermal conductivity in (Table 1). These differences have a significant effect on the behavior of the materials during explosive welding.

**Figure 1:**
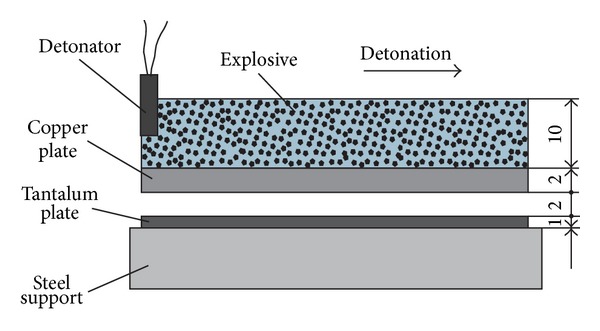
Schematic diagram of explosive welding with parallel arrangement of copper and tantalum plates.

Explosive welding of copper and tantalum plates arranged in parallel (Figure 1) was performed at the Lavrent'ev Institute of Hydrodynamics. A tantalum plate 1 mm thick was placed on a steel support. The flyer plate was a 2 mm copper plate. The distance between the copper and tantalum plates was set equal to 2 mm. The explosive was 6 ZhV ammonite of density 0.9 g·cm^3^, which was placed directly on the copper plate. The detonation velocity of the explosive was 3800 m·s^−1^, and the plate collision angle was 17°. Prior the welding the surfaces of the plates were grinded using SiC abrasive paper (320 grit).

The thermal stability of the Cu-Ta joint was determined by annealing samples at temperatures in the range of 100 to 900°C. The dwell time in the furnace was 1 h. A characteristic feature of tantalum is its active interaction with the gases that make up the air. For this reason, the welded joints were annealed in a vacuum furnace at 10^-6 ^Pa.

Structural studies were performed on transverse sections. For metallographic studies and microhardness measurements, laminated composite specimens were cut along the direction of shock-wave propagation. Metallographic sections were prepared by conventional techniques comprising grinding and polishing. The structure of copper was revealed by etching in an aqueous solution of ferric chloride and hydrochloric acid.

Metallographic studies were carried out using a Carl Zeiss Axio Observer Z1m microscope. Subtle structural changes in plastically deformed materials were studied using a Tecnai G2 20 TWIN transmission electron microscope. The objects of study were foils. The foils were prepared by a technique combining cutting of samples with an electric spark machine, mechanical thinning to a thickness of 100 *μ*m, dimple grinding using a Gatan Dimple Grinder, and final ion thinning in a Gatan PIPS 659 ion mill. The structure of the sample material in the region of interaction was studied using an EVO 50 XVP scanning electron microscope.

Microhardness of the samples was measured before and after heat treatment with a Wolpert Group 402 MVD microhardness tester. The load on the diamond indenter was 0.245 N. The measurement procedure involved the creation of a track of indentations perpendicular to the weld in the direction from the copper layer to the tantalum layer. The indenter indentation were spaced at 50 *μ*m intervals.

## 3. Results and Discussion

### 3.1. Structural Analysis of the Explosively Welded Cu-Ta Joint

**Figure 2:**
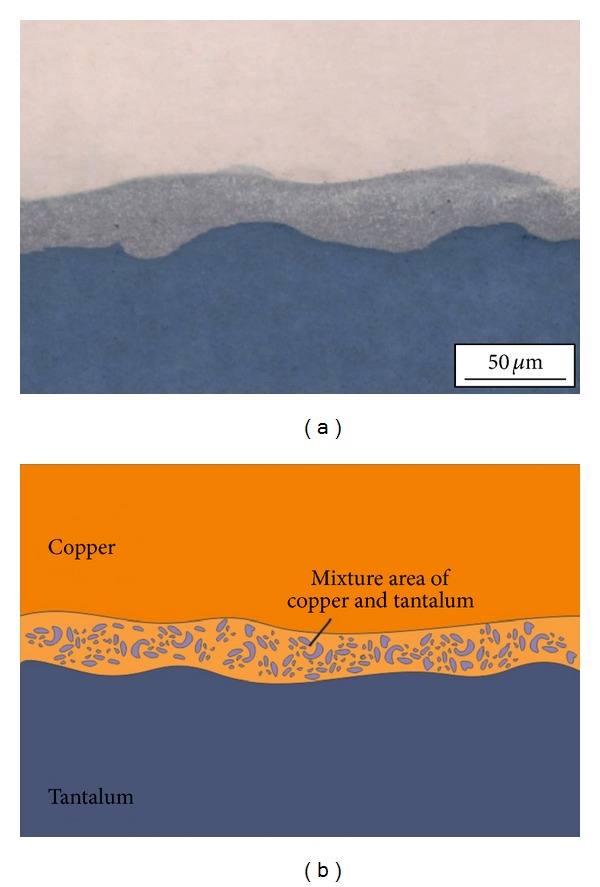
General view (a) and schematic diagram (b) of Cu-Ta joint produced by explosive welding.

The structure and the scheme of the explosively welded Cu-Ta joint in a longitudinal section are shown in Figure 2. The line of junction of the plates obtained under the welding conditions described in Section 2 does not have a regular wavy shape that is characteristic of many explosively welded metals. Formation of a wavy junction is prevented by the large differences in strength properties, density, and melting point between copper and tantalum. Optical metallography and scanning electron microscopy identified a continuous layer 20 ⋯ 40 *μ*m thick between the copper and tantalum plates. The layer contains the material formed by mixing of the dissimilar materials under dynamic interaction. During the formation of wavy welds, such structure of a mixture of materials is observed only in the vortex zones formed near the crests and troughs of individual waves.

Due to the high degree of dispersion of the resulting mixture, it is difficult to determine the size and shape of individual elements of the structure in the layer by scanning electron microscopy. For this reason, a more detailed structural analysis of the mixing regions was performed using transmission electron microscopy. The main factors determining the structural features of the material are the high rates and amounts of strain in the surface layers of the plates, high heating temperatures of tantalum and copper in the contact area, and the presence of a cumulative sheet of fine particles of the interacting materials.

**Figure 3:**
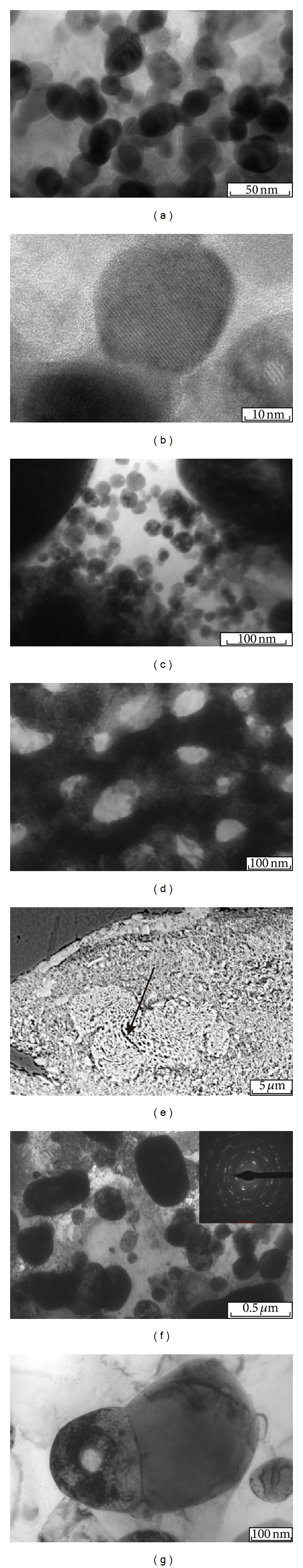
Structure of the intermediate layer with a heterophase structure after explosive welding of copper and tantalum plates (a–e) and after heating for 1 hour at 900°C (f, g). (e) is a scanning electron micrograph, and the other images are transmission electron micrographs.

In general, the structure of the intermediate heterophase layer can be defined as a mixture of fine copper and tantalum. The matrix material in the layer, characterized by continuity, is predominantly copper. A lot of dark rounded particles of tantalum can be seen against the background of the bright copper matrix. Tantalum particles in copper have sizes of 5 ⋯ 500 nm, with a predominance of 5 ⋯ 50 nm particles (Figures 3(a) and 3(b)). An example of a combination of a finely dispersed structure with larger particles of tantalum is presented in Figure 3(c).

In addition, the heterophase layer was experimentally found to contain microvolumes in which the matrix material is tantalum and copper is in the form of separate islands. A transmission electron micrograph of a structure of this type is shown in Figure 3(d). The copper particle size is ~50 ⋯ 100 nm.  Figure 3(e) shows a scanning electron micrograph of the structure of the intermediate layer. The microvolume of tantalum with copper particles is indicated by the arrow.

**Figure 4:**
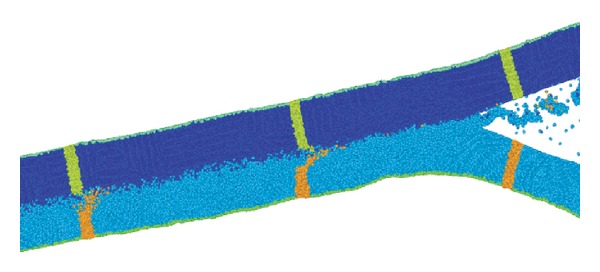
Direction of the jet in the collision of a copper plate (top) and an aluminum plate (bottom) simulated using a molecular dynamics model [14].

**Figure 5:**
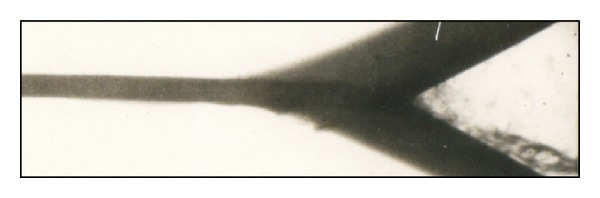
X-ray photograph of copper plate colliding with different velocities (the upper plate at a velocity *ν*_*n*2_ = 1.26 km/s and the lower plate at a velocity *ν*_*n*1_ = 1.12 km/s) [15].

The mechanism of formation of the intermediate heterophase layer can be described as follows (Figures 4 and 5). During dynamic interaction of copper and tantalum plates, a discrete cumulative jet consisting mainly of fragments of copper is formed at the point of contact. It is known [14, 15] that if the colliding metals differ greatly in density or velocity, the discrete jet does not move along the bisector of the collision angle but is deflected toward the denser (in our case, tantalum) or slow-moving plate.

It should be emphasized that the surface of the joined plates is rough. The size of the majority of particles in the gap between the joined plates (5 ⋯ ~50 nm) is 2 ⋯ 3 orders of magnitude smaller than the roughness values of the metal workpieces. Thus, some of the highly dispersed particles moving faster than the point of contact >3800 m·s^−1^ penetrate into the rough surface layers of the tantalum plate.

**Figure 6:**
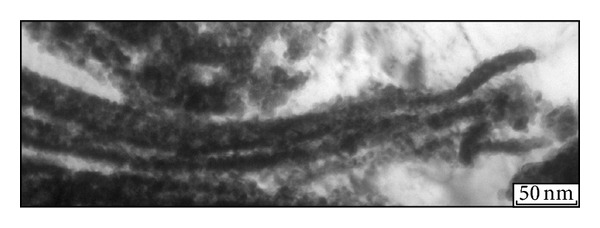
Banded clusters of tantalum in copper.

The impact of the jet fragments on the surface layers of the tantalum plate leads to the formation of a mixing zone and a cloud of fine particles of a mixture of copper and tantalum. Most of the tantalum particles in the mixing zone are randomly distributed in copper. Note that the melting points of copper and tantalum differ by almost a factor of three (1084°C and 2996°C). Tantalum particles remain in the solid state under such collisions. At the same time, microvolumes of copper with ordered arrangement of tantalum particles are also observed. The tantalum clusters shown in Figure 6 have a banded form. Structural analysis performed at higher magnifications indicates that the elongated structures observed in the micrographs consist of individual nanosized particles of tantalum. It can be suggested that their formation results from the flow of a discrete copper jet on a rough surface of tantalum, whose fragments are stretched by the high-velocity flow.

### 3.2. Thermal Stability of the Cu-Ta Welded Joint

The thermal stability of the Cu-Ta joint was evaluated by heating the samples with subsequent analysis of structural changes and microhardness of the materials. The heating temperature was in the range of 100 ⋯ 900°C. The exposure at each temperature was 1 h.

**Figure 7:**
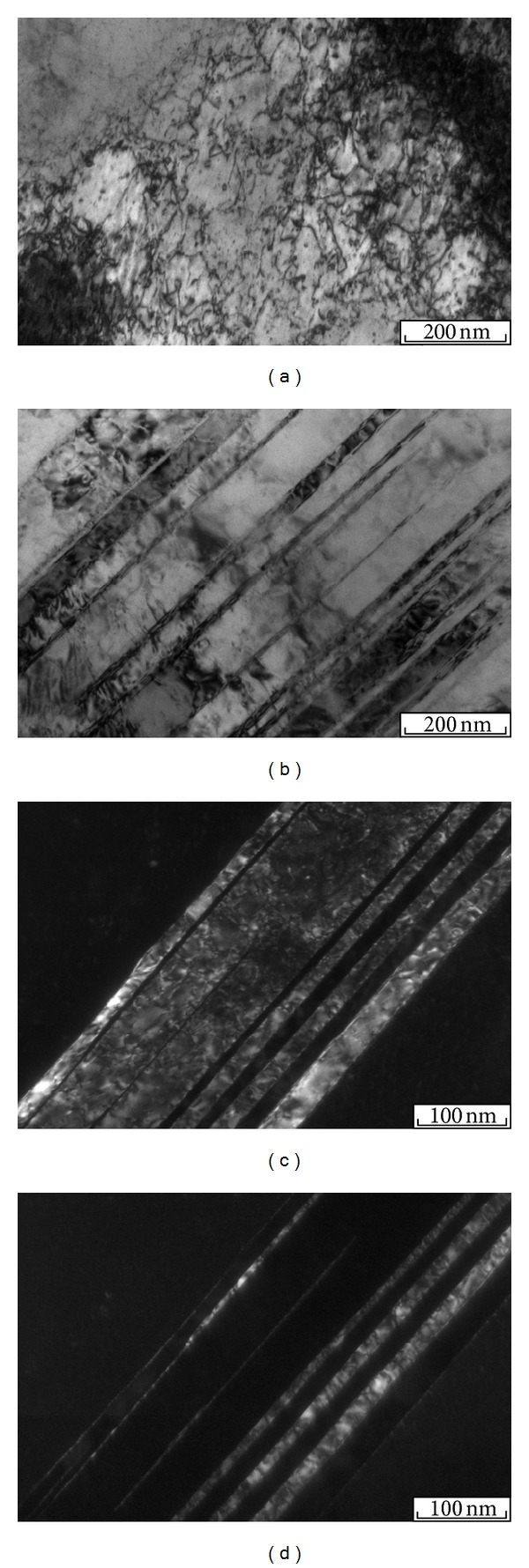
Dislocation structure (a) and twins of deformation origin (b–d) in a copper plate at a distance of 50 *μ*m from the intermediate layer. (b) bright-field and (c, d) dark-field transmission electron micrographs of the same section.

Increase in the heating temperature was accompanied by structural transformations, leading to strength degradation of the copper and tantalum plates and the heterophase layer located between them. Structural analysis revealed the most pronounced changes in the copper plate. In the initial state (before welding), the grain size was 22 *μ*m. Dynamic interaction between the plates leads to deformation strengthening of copper in regions directly adjacent to the weld. This is indicated by the formation of microvolumes with increased dislocation density (Figure 7(a)) and a set of twins of deformational origin. Typically, twins ~10 ⋯ 50 nm wide are arranged in the form of individual stacks (Figure 7(b)). Figures 7(b)–7(d) shows bright- (b) and dark-field images of the same region of plastically deformed copper. Twinning deformation is typical of metals under high-velocity deformation, including explosive welding [16, 17].

The most notable changes in the strength properties of the plastically deformed materials occur upon heating to values close to the recrystallization temperature. After heating to 500°C, the copper plate has no indications of recrystallization. During heating to 600°C, the recrystallization of copper is nonuniform. Recrystallization occurs in islands spaced at 2000 *μ*m intervals along the surface of junction of the plates. It should be noted that in the copper layer, strain far exceeding the critical value occurs at a depth of several tens of micrometers. In the rest of the volume of the copper plate, the amount of strain is close to the critical value. For this reason, copper recrystallization is accompanied by a sharp increase in the grain size. After heating to 700°C, the grain size in the recrystallized zones is 440 *μ*m. In the samples exposed at 900°C for 1 h, the grain size increased to 620 *μ*m.

**Figure 8:**
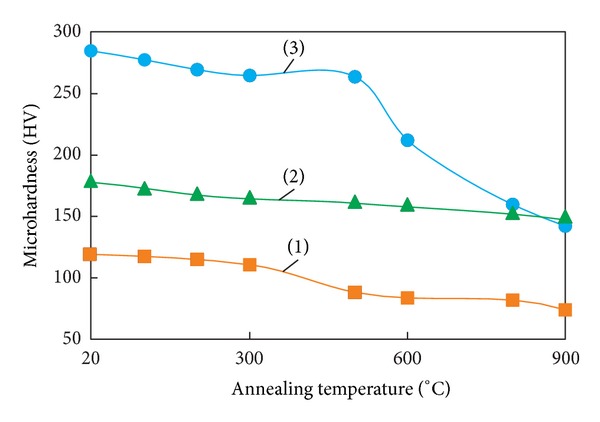
Effect of heating temperature on the microhardness of copper (1), tantalum (2), and the intermediate layer with a heterophase structure (3).

**Figure 9:**
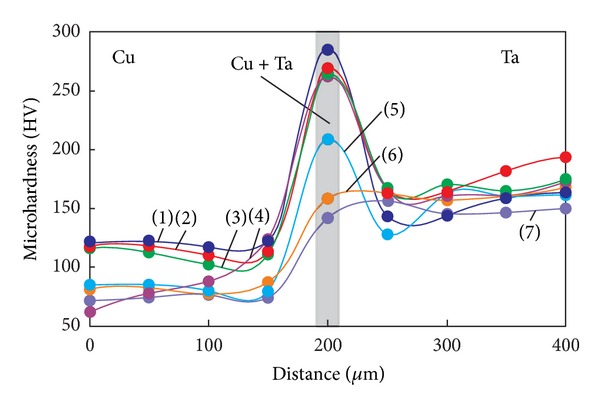
Microhardness of the intermediate layer and its adjacent regions after explosive welding of copper and tantalum plates and subsequent heating of the weld at different temperatures. (1) without heat treatment, (2) heating to 200°C, (3) 300°C, (4) 500°C, (5) 600°C, (6) 800°C, and (7) 900°C.

Results of microhardness measurements of thermally untreated samples of welded joints and those annealed at various temperatures are presented in Figures 8 and 9. Microhardness was measured in a direction perpendicular to the welded joint within 200 *μ*m on each side of the plane of junction of the plates. The highest microhardness values were recorded for thermally untreated welded joints. After explosive welding, the microhardness of copper was ~130 HV, and that of tantalum as ~160 HV. Maximum microhardness (280 HV) was recorded in the narrow intermediate layer of heterophase structure.

The observed effect of hardening of the material is probably due to the formation of a highly dispersed mixture and strain hardening of tantalum. Despite the large amount of plastic strain in the surface layer of the copper plate, strain hardening does not play a significant role in this case. Rapid heating of local microvolumes of the materials leads to melting of copper and eliminates the dislocations structure formed in the zone of dynamic interaction of the plates. The strain hardening effect is eliminated not only by melting but also upon reaching a temperature leading to recrystallization processes. These processes are typical of explosive welding of metallic materials [17–19]. During welding of carbon and alloy steels, a sharp increase in the strength properties in the weld zones is due to the hardening mechanism and the formation of a martensitic structure. In the Cu-Ta system, with no mutual solubility of the elements, this mechanism cannot lead to an increase in the microhardness of the material in the weld zone.

In samples heated to 500°C, the microhardness in the zone of mixing of tantalum and copper does not change significantly. This indicates that the heterophase structure is thermally stable in this temperature range. Heating to 600°C or above results in a marked decrease in the microhardness of the material. After annealing at 900°C, the microhardness of the intermediate layer with mixed structure becomes equal to the microhardness of the tantalum plate (~150 HV). In contrast to this layer, the temperature dependence corresponding to tantalum shows no changes. This is due to the fact that the recrystallization temperature of tantalum is more than 300°C higher than the maximum temperature of annealing of the welded joint. Increasing the annealing temperature of copper to 900°C leads to an almost twofold decrease in its microhardness (from 130 to 75 HV) due to relaxation processes in dynamically deformed zones and the formation of a more equilibrium structure than that of the initial one [20].

## 4. Conclusions

During explosive welding in the joint area of tantalum and copper, plates form an intermediate layer which has a heterophase structure and consist of a mixture of fragments of dissimilar materials. The matrix material is predominantly copper. Tantalum in copper is in the form of isolated particles. Less common are microvolumes in which the matrix is tantalum with copper particles embedded in it. Based on the sizes of the dispersed-phase particles observed in the intermediate layer, the material can be classified as a highly dispersed system. The size of tantalum particles is predominantly in the nanometer range (~5 ⋯ 50 nm), which corresponds to the size range of colloidal particles. A mechanism for the formation of this structure was proposed which involves the formation of a fragmented, mainly copper, cumulative jet ahead of the point of contact of the plates. The jet moves directly on the surface of the tantalum plate and interacts with the roughness of the latter. The Cu-Ta mixing area is thermally stable upon heating to 500°C. Annealing at 900°C leads to a decrease in the microhardness of the intermediate layer with the heterophase structure from 280 HV to 150 HV.

## Figures and Tables

**Table 1:** Some properties of copper and tantalum.

Features	Cu	Ta
Crystal lattice	fcc	bcc
Density, g/cm^3^	8,93	16,6
Melting point, °C	1084	2996
Recrystallization temperature, °C	300	1300
Thermal conductivity, W/m·K	390	52.1
